# Rivaroxaban vs. enoxaparin for preventing venous thromboembolism after hip fracture operations: a retrospective cohort study

**DOI:** 10.3389/fsurg.2025.1483611

**Published:** 2025-05-09

**Authors:** Xiaofeng Zheng, Lifu Wang, Yinhua Chen, Lijuan Liu, Zijiao Zhang, Cong Xiao

**Affiliations:** Department of Orthopedics, The Third Hospital of Mianyang, Sichuan Mental Health Center, Mianyang, China

**Keywords:** hip fractures, venous thromboembolism, rivaroxaban, enoxaparin, cost

## Abstract

**Introduction:**

Emerging evidence suggests that rivaroxaban may be effective in preventing venous thromboembolism (VTE) in patients with orthopedic trauma, resulting in fewer bleeding complications. This study aimed to evaluate the efficacy and safety of rivaroxaban compared with enoxaparin in preventing VTE in patients undergoing hip fracture surgery.

**Materials and methods:**

This single-center, retrospective cohort study included patients who received either oral rivaroxaban or subcutaneous injections of enoxaparin for VTE prophylaxis following hip fracture surgery from 2020 to 2023. The data obtained included patient demographics, fracture classification, time to surgery, and procedures performed. The main outcomes assessed were the incidence of VTE and hemorrhagic events and death within 30 days of surgery. The daily costs of the two types of medications were also recorded.

**Results:**

A total of 166 patients were included. The incidence of VTE was 9.5% in the rivaroxaban group and 26.61% in the enoxaparin group. Hemorrhagic events occurred in 9.52% and 1.61% of patients in the rivaroxaban and enoxaparin groups, respectively. No deaths occurred in either group. The average daily cost of rivaroxaban was 26.49 ± 4.77 Chinese yuan, while that of enoxaparin was 75.24 ± 18.54 Chinese yuan.

**Conclusion:**

In this cohort study, rivaroxaban was found to be significantly more effective than enoxaparin in reducing postoperative VTE after hip fracture, but it was associated with a higher risk of hemorrhagic events. Additionally, the average daily cost of rivaroxaban was lower. To identify patients who will derive the maximum advantages from this treatment, larger prospective studies are needed.

## Introduction

Venous thromboembolism (VTE), including pulmonary embolism (PE) and deep vein thrombosis (DVT), is a significant cause of morbidity and mortality subsequent to hip fractures (1). In the absence of preventive measures, the incidence of VTE after hip fracture is estimated to reach 16.6% (2). The perioperative incidence of VTE in patients with hip fracture can be reduced by implementing effective anticoagulation strategies, typically involving the utilization of anticoagulants such as low-molecular-weight heparin (LMWH) (3), aspirin (4), or warfarin (3).

In recent years, the utilization of direct oral anticoagulants (DOACs) in orthopedic surgery has significantly expanded (3, 5, 6). DOACs offer advantages due to their oral administration, predictable pharmacological profile, and lack of required monitoring. Moreover, multiple studies have demonstrated that compared with conventional anticoagulants, DOACs are associated with lower rates of VTE without an increased risk of hemorrhage (7, 8). However, the evidence supporting the use of rivaroxaban for VTE prophylaxis following hip fracture surgery is limited, with LMWH being the preferred form of anticoagulation (9, 10). This study aimed to compare rivaroxaban with enoxaparin for preventing VTE in terms of effectiveness, safety, and cost in patients undergoing hip fracture surgery.

## Methods and materials

### Population

This single-center, retrospective, cohort study included patients who received either rivaroxaban or enoxaparin for VTE prophylaxis following hip fracture surgery from 1 January 2020 to 31 December 2022.

The inclusion criteria were as follows: participants were ≥18 years of age, had a confirmed diagnosis of either femoral neck or intertrochanteric femoral fractures, and received rivaroxaban anticoagulant therapy or enoxaparin anticoagulant therapy during the perioperative period.

Patients with any of the following characteristics were excluded: missing data, previous PE or DVT, previous major hemorrhage, underlying malignancy, or inherited coagulation disorder. All patients were followed up for 30 days postoperatively.

### Clinical parameters

The following data were obtained: patient demographics, fracture classification, American Society of Anesthesiologists (ASA) classification, time to surgery, surgical procedures, and hospital stay. The outcomes assessed were the incidence of PE or DVT and hemorrhagic events and death within 30 days of surgery. PE was diagnosed using computed tomography pulmonary angiography, and DVT was diagnosed using duplex ultrasound scanning. Each patient received a postoperative lower limb venous ultrasound examination. Computed tomography pulmonary angiography was performed for patients with symptomatic PE. Hemorrhagic events include major bleeding, clinically relevant non-major bleeding (CRNMB), and minor bleeding. Major bleeding was defined as a decrease in the hemoglobin level of ≥2 g dl^−1^, bleeding leading to transfusion, bleeding at a critical site, or bleeding contributing to death (11). CRNMB was defined as bleeding not meeting the criteria for major bleeding but associated with medical intervention, temporary cessation of study treatment, patient discomfort such as pain, or impairment of activities of daily living (12). Minor bleeding was defined as bleeding not meeting the criteria for either major bleeding or CRNMB.

In the event of death, the coroner's report, hospital, and general practice records were reviewed to establish the cause of death. During the study, the cost of rivaroxaban or enoxaparin was also recorded.

### Administration regime

Rivaroxaban (Xarelto, Bayer) was administered at 10 mg qd beginning 6–10 h after surgery. Enoxaparin (Clexane, Sanofi) was administered via subcutaneous injection at a dosage of 4,000 IU qd beginning 6 h after surgery. The treatment duration for both groups was set to a standardized period of 30 days.

### Statistical analysis

Multivariate analysis was performed using GraphPad Prism version 8.0.0 for Windows (GraphPad Software, San Diego, CA, USA). The relevant data are expressed as percentages, means, and standard deviations. Parametric data were analyzed via Student's *t*-test, whereas categorical data were assessed using the chi-square test. The presentation of relative risk (RR) includes a 95% confidence interval (CI) and *P*-value. Power analysis was also performed (13).

### Ethical approval

This study was conducted retrospectively, utilizing anonymous data that had been previously collected during patient assessments or for service evaluation. The local ethics committee reviewed the study proposal and determined that ethical approval was not required.

## Results

This retrospective cohort study included 166 patients, with 42 patients receiving rivaroxaban treatment and 124 patients receiving enoxaparin treatment. All participants were closely monitored for 30 days as part of our final analysis.

The mean age of the patients in the rivaroxaban group was 77.95 ± 12.91 years, and the mean age of those in the enoxaparin group was 76.27 ± 11.00 years; no significant difference was found between the two groups. The male–female ratio was 18:24 in the rivaroxaban group and 56:68 in the enoxaparin group. There were no significant differences in sex composition between the two groups.

The enoxaparin group included 41 patients with femoral neck fractures, 81 patients with intertrochanteric fractures, and 2 patients with subtrochanteric fractures. Proximal femoral nail antirotation (PFNA) was performed in 82 patients, accounting for 66.13% of all patients. This was followed by total hip arthroplasty (THA) in 41 patients and the use of the femoral neck system (FNS) in 1 patient. The mean time to surgery was 2.24 ± 0.93 days. The rivaroxaban group included 18 patients with femoral neck fractures and 24 patients with intertrochanteric fractures. PFNA was performed in 24 patients. This was followed by THA in 18 patients. The mean time to surgery was 2.37 ± 1.53 days. The baseline demographic and clinical characteristics are presented in Table 1.

**Table 1:** Baseline demographic and clinical characteristics.

Characteristic	Rivaroxaban, *n* = 42	Enoxaparin, *n* = 124	*P*-value
Age, years	77.95 ± 12.91	76.27 ± 11.00	0.42
Gender			0.86
Male	24	68	
Female	18	56	
ASA Scores			0.79
ASA I	0	0	
ASA II	18	57	
ASA III	24	69	
ASA IV	0	0	
Fracture classification			0.10
Garden III	15	38	
Garden IV	1	3	
Russell–Taylor IA	1	1	
Russell–Taylor IIA	0	1	
Evans–Jensen II	1	4	
Evans–Jensen III	7	39	
Evans–Jensen IV	2	20	
Evans–Jensen V	11	16	
Evans–Jensen R	1	0	
Surgery procedure			0.001
Total hip arthroplasty	23	41	
FNS	3	1	
PFNA	16	82	
Time to surgery, days	2.24 ± 0.93	2.37 ± 1.53	0.59

ASA, American Society of Anesthesiologists.

The rivaroxaban group had a VTE incidence of 9.52% (95% CI: 0.04–0.22), that of the enoxaparin group was 26.61% (95% CI: 0.19–0.35), and the RR was 0.36 (95% CI: 0.14–0.87). The incidence of VTE in the rivaroxaban group was significantly lower than that in the enoxaparin group (*P* < 0.05), and the statistical power was approximately 81%. PE was not observed in either group (*P* > 0.05).

Hemorrhage occurred in 9.53% (95% CI: 0.04–0.22) of patients in the rivaroxaban group and 1.61% (95% CI: 0.01–0.06) of patients in the enoxaparin group (RR: 5.90, 95% CI: 1.29–26.76, *P* = 0.017). No major bleeding was observed in either group. The statistical power was approximately 53.9%.

The average daily cost of rivaroxaban was 26.49 ± 4.77 Chinese yuan, whereas that of enoxaparin was 75.24 ± 18.54 Chinese yuan. The average daily cost of rivaroxaban was lower than that of enoxaparin (*P* < 0.05), and the statistical power was approximately 100%. The efficacy and safety outcomes are shown in Table 2.

**Table 2:** Efficacy and safety outcomes in all patients.

Outcomes	Rivaroxaban, *n* = 42	Enoxparin, *n* = 124	*P*-value
All venous thromboembolism	4 (9.52%, 95% CI: 0.04–0.22)	33 (26.61%, 95% CI: 0.19–0.35)	0.02
Pulmonary embolism	0	0	
Deep venous thrombosis	4 (9.52%, 95% CI: 0.04–0.22)	33 (26.61%, 95% CI: 0.19–0.35)	0.02
Hemorrhagic events	4 (9.52%, 95% CI: 0.04–0.22)	2 (1.61%, 95% CI: 0.004–0.06)	0.017
Major bleeding	0	0	
CRNBM^a^	2 (4.76%, 95% CI: 0.01–0.16)	1 (0.8%, 95% CI: 0.001–0.04)	0.17
Minor bleeding	2 (4.76%, 95% CI: 0.01–0.16)	1 (0.8%, 95% CI: 0.001–0.04)	0.17
Cost daily (Chinese yuan)	26.49 ± 4.77	75.24 ± 18.54	<0.0001
All-cause mortality	0 (0%)	0 (%)	>0.99

^a^ CRNMB, clinically relevant non-major bleeding.

## Discussion

In this study, the administration of rivaroxaban for preventing VTE after hip fracture surgery demonstrated a lower incidence of VTE than did the administration of enoxaparin. The use of rivaroxaban for the prevention of postoperative VTE in patients with lower limb fractures is controversial. Some studies suggest that DOACs exhibit comparable efficacy to LMWH in the prevention of VTE following lower limb orthopedic surgery (14–18). In these studies, the results of direct comparisons between rivaroxaban and enoxaparin indicate that both have similar efficacy in preventing venous thromboembolism (16, 17). However, the results of this study are quite different because rivaroxaban produced a reduced incidence of VTE. These findings are similar to those of previous evaluations of the effectiveness of rivaroxaban in preventing VTE in patients undergoing lower limb orthopedic surgery, where the incidence of VTE was lower in patients treated with rivaroxaban than in those treated with enoxaparin (19–21). Although the effectiveness of rivaroxaban and enoxaparin is debated, existing studies have consistently shown that rivaroxaban is not inferior to enoxaparin in preventing VTE.

The incidence rates of VTE in the rivaroxaban and enoxaparin groups were 9.52% (95% CI: 0.04–0.22) and 26.61% (95% CI: 0.19–0.35), respectively, which were higher than those reported in other studies (22). In a study conducted by Tang et al. (10), the incidence of VTE was found to be significantly lower with the administration of rivaroxaban (5.2%) than with the administration of enoxaparin (14.7%). Differences in study design may account for the variation in rates of VTE reported in both the rivaroxaban and enoxaparin groups in the present study. Patients participating in randomized controlled trials (RCTs) are subjected to regular examinations for VTE, which can result in the identification of patients with asymptomatic PE and DVT. Although our study was not an RCT but rather a retrospective cohort study, our department ensured the safety of patients by conducting postoperative VTE examinations for all patients undergoing lower limb orthopedic surgery. This ultimately led to a higher incidence of VTE, as more patients with asymptomatic VTE were discovered.

Another reason might be related to compliance. One study (23) investigated the association between patient compliance and the efficacy of LMWH in patients undergoing hip fracture surgery and receiving anticoagulation treatment. This study of 1,214 patients with hip fractures categorized participants into three compliance groups: low (<14 days, 64.7%), normal (14–27 days, 19.0%), and high (≥28 days, 16.3%). Postoperative VTE incidence significantly differed across groups, at 9.6%, 5.4%, and 4.2%, respectively (*P* = 0.013). Multivariate analysis revealed that low compliance (<14 days) was an independent risk factor for VTE (OR = 2.77, 95% CI: 1.27–6.04). This study further emphasized that compliance plays a critical role in influencing VTE incidence and that low compliance with treatment can increase the incidence of VTE. Bergqvist et al. (24) studied compliance with VTE prevention therapy in patients with hip fractures undergoing total hip arthroplasty and knee arthroplasty. The results showed that treatment via injection negatively affected patient compliance. Furthermore, insufficient basic healthcare institutions and limited family care have also contributed to the low compliance of patients in China (25).

Another reason for the higher incidence of VTE in the enoxaparin group might be related to the dose. To treat thromboprophylaxis, the administration of enoxaparin at a fixed daily dose of 4,000 IU is recommended (26). While standard doses can be used in most patients, patients at the extremes of weight are at risk for either overdosing (low-body-weight patients) or underdosing (high-body-weight patients) (27, 28).

The mortality rate for both groups of patients in this study was zero. This was mainly due to the improved detection and subsequent treatment of VTE in postsurgical patients, which helped prevent the occurrence of PE. Both DVT and PE are associated with potentially significant morbidity and mortality (29).

The incidence rates of hemorrhage in the rivaroxaban and enoxaparin groups were 9.53% and 1.61%, respectively. Four patients in the rivaroxaban group and two patients in the enoxaparin group experienced hemorrhagic events. No patients in either group experienced any major bleeding. The incidence of minor bleeding in the rivaroxaban group was significantly greater than that in the enoxaparin group. However, the statistical power was only 59.6%, indicating that the sample size was insufficient to reliably detect the observed difference. This trend toward a potential risk of hemorrhage has not been reported in earlier studies of elective orthopedic surgery (10, 22).

Most patients with hip fractures are elderly and physically weak and have multiple comorbidities, making them prone to fatal hemorrhage. Although no major bleeding was observed in this study, further research is warranted to investigate this situation.

While LMWH must be administered via subcutaneous injection due to its pharmaceutical properties, this administration method may lead to low patient compliance post-discharge and to challenges in completing the entire anticoagulation treatment course (30).

In contrast, oral administration is the method of administration for rivaroxaban. The enhanced convenience and the fact that elderly patients do not require coagulation function monitoring and dose adjustment may help improve compliance with long-term anticoagulant therapy after discharge.

Hence, there is a greater probability that individuals will adhere to their healthcare provider's guidance and maintain the use of rivaroxaban for VTE prevention, potentially resulting in earlier discharge. In addition, rivaroxaban is less expensive than enoxaparin. The average daily cost of rivaroxaban in the present study was lower than that of enoxaparin (26.49 ± 4.77 vs 75.24 ± 18.54 Chinese yuan, respectively).

Rivaroxaban not only is a more affordable medication but also requires less care time and hospital resources for management. In Australia, the cost of a course of rivaroxaban post-THA is $99.6, whereas that of enoxaparin is $151.1 (31). According to calculations using data from the American healthcare system, using rivaroxaban instead of enoxaparin can reduce treatment costs by $262 per patient (32).

The effectiveness of rivaroxaban makes it a cost-effective drug for the prevention of VTE, despite the potential risk of minor bleeding.

Our research has several limitations. First, due to the retrospective study design, there were limitations in terms of patient randomization and control. Senior clinical doctors decide whether to prescribe rivaroxaban based on the presence of risk factors for thromboembolism and consider the bleeding risk. Therefore, the rivaroxaban group in this study is likely to represent a selected group of patients who are likely to benefit from this treatment. Our study was limited by a sample size of 166 patients, and the sample size was not estimated in advance. A larger sample size is needed to detect clinically significant differences between the two groups. Because enoxaparin is still one of the first-line treatments for VTE prevention in China, only a small number of patients receive rivaroxaban.

## Conclusion

In this retrospective cohort study, rivaroxaban was found to be more effective than enoxaparin in reducing the risk of VTE after hip fracture surgery. However, a trend toward an increased risk of hemorrhage was noted, which necessitates further investigation. However, we discovered that the cost-effectiveness of rivaroxaban, considering its effectiveness in reducing VTE, surpassed that of enoxaparin. Therefore, after the patients most likely to benefit from this treatment are identified and the potential risk of bleeding is reduced, rivaroxaban may be an economically effective and safe alternative for routine thromboprophylaxis following hip fracture surgery. Future studies involving a larger number of patients are necessary to determine which patients are most likely to benefit from this treatment.

## Data Availability

The raw data supporting the conclusions of this article will be made available by the authors, without undue reservation.

## References

[1] Todd CJ Freeman CJ Camilleri-Ferrante C Palmer CR Hyder A Laxton CE Differences in mortality after fracture of hip: the east Anglian audit. Br Med J. (1995) 310(6984):904–8. 10.1136/bmj.310.6984.904 7719180 PMC2549290

[2] Wang T Wang T Guo J Long Y Yin Y Hou Z. Risk factors for preoperative deep venous thrombosis in hip fracture patients: a meta-analysis. J Orthop Traumatol. (2022) 23(1):19. 10.1186/s10195-022-00639-6 35391566 PMC8991371

[3] Trivedi NN Sivasundaram L Wang C Kim CY Buser Z Wang JC Chemoprophylaxis for the hip fracture patient: a comparison of warfarin and low-molecular-weight heparin. J Orthop Trauma. (2019) 33(5):216–9. 10.1097/BOT.0000000000001435 31008818

[4] Huang Q Xing S Zeng Y Si H Zhou Z Shen B. Comparison of the efficacy and safety of aspirin and rivaroxaban following enoxaparin treatment for prevention of venous thromboembolism after hip fracture surgery. Orthop Surg. (2019) 11(5):886–94. 10.1111/os.12542 31663285 PMC6819168

[5] Nederpelt CJ Breen KA el Hechi MW Krijnen P Huisman MV Schipper IB Direct oral anticoagulants are a potential alternative to low-molecular-weight heparin for thromboprophylaxis in trauma patients sustaining lower extremity fractures. J Surg Res. (2021) 258:324–31. 10.1016/j.jss.2020.10.009 33187673

[6] Fuji T Fujita S Kawai Y Nakamura M Kimura T Kiuchi Y Safety and efficacy of edoxaban in patients undergoing hip fracture surgery. Thromb Res. (2014) 133(6):1016–22. 10.1016/j.thromres.2014.03.009 24680549

[7] Cheng JW Barillari G. Non-vitamin K antagonist oral anticoagulants in cardiovascular disease management: evidence and unanswered questions. J Clin Pharm Ther. (2014) 39(2):118–35. 10.1111/jcpt.12122 24383983

[8] Eriksson BI Quinlan DJ Eikelboom JW. Novel oral factor Xa and thrombin inhibitors in the management of thromboembolism. Annu Rev Med. (2011) 62:41–57. 10.1146/annurev-med-062209-095159 21226611

[9] Zhang C Xu B Liang G Zeng X Yang C Zhang F Rivaroxaban versus nadroparin for preventing deep venous thrombosis after total hip arthroplasty following femoral neck fractures: a retrospective comparative study. J Int Med Res. (2018) 46(5):1936–46. 10.1177/0300060518762281 29560772 PMC5991221

[10] Tang Y Wang K Shi Z Yang P Dang X. A RCT study of rivaroxaban, low-molecular-weight heparin, and sequential medication regimens for the prevention of venous thrombosis after internal fixation of hip fracture. Biomed Pharmacother. (2017) 92:982–8. 10.1016/j.biopha.2017.05.107 28605879

[11] Schulman S Angerås U Bergqvist D Eriksson B Lassen MR Fisher W. Definition of major bleeding in clinical investigations of antihemostatic medicinal products in surgical patients. J Thromb Haemost. (2010) 8(1):202–4. 10.1111/j.1538-7836.2009.03678.x 19878532

[12] Kaatz S Ahmad D Spyropoulos AC Schulman S. Definition of clinically relevant non-major bleeding in studies of anticoagulants in atrial fibrillation and venous thromboembolic disease in non-surgical patients: communication from the SSC of the ISTH. J Thromb Haemost. (2015) 13(11):2119–26. 10.1111/jth.13140 26764429

[13] Glueck DH. Sample size calculations in clinical research 2nd edition by Chow, S.-C., Shao, J., and Wang, H. Biometrics. (2008) 64(4):1307–8. 10.1111/j.1541-0420.2008.01138_10.x

[14] Lassen MR Raskob GE Gallus A Pineo G Chen D Hornick P. Apixaban versus enoxaparin for thromboprophylaxis after knee replacement (ADVANCE-2): a randomised double-blind trial. Lancet. (2010) 375(9717):807–15. 10.1016/S0140-6736(09)62125-5 20206776

[15] Lassen MR Gallus A Raskob GE Pineo G Chen D Ramirez LM. Apixaban versus enoxaparin for thromboprophylaxis after hip replacement. N Engl J Med. (2010) 363(26):2487–98. 10.1056/NEJMoa1006885 21175312

[16] Eriksson BI Borris LC Friedman RJ Haas S Huisman MV Kakkar AK Rivaroxaban versus enoxaparin for thromboprophylaxis after hip arthroplasty. N Engl J Med. (2008) 358(26):2765–75. 10.1056/NEJMoa0800374 18579811

[17] Lassen MR Ageno W Borris LC Lieberman JR Rosencher N Bandel TJ Rivaroxaban versus enoxaparin for thromboprophylaxis after total knee arthroplasty. N Engl J Med. (2008) 358(26):2776–86. 10.1056/NEJMoa076016 18579812

[18] Eriksson BI Dahl OE Rosencher N Kurth AA van Dijk CN Frostick SP Dabigatran etexilate versus enoxaparin for prevention of venous thromboembolism after total hip replacement: a randomised, double-blind, non-inferiority trial. Lancet. (2007) 370(9591):949–56. 10.1016/S0140-6736(07)61445-7 17869635

[19] Turpie AG Lassen MR Davidson BL Bauer KA Gent M Kwong LM Rivaroxaban versus enoxaparin for thromboprophylaxis after total knee arthroplasty (RECORD4): a randomised trial. Lancet. (2009) 373(9676):1673–80. 10.1016/S0140-6736(09)60734-0 19411100

[20] Zou Y Tian S Wang Y Sun K. Administering aspirin, rivaroxaban and low-molecular-weight heparin to prevent deep venous thrombosis after total knee arthroplasty. Blood Coagul Fibrinolysis. (2014) 25(7):660–4. 10.1097/MBC.0000000000000121 24695091

[21] Huang HF Li S-S Yang X-T Xie Q Tian X-B. Rivaroxaban versus enoxaparin for the prevention of venous thromboembolism after total knee arthroplasty: a meta-analysis. Medicine (Baltimore). (2018) 97(48):e13465. 10.1097/MD.0000000000013465 30508972 PMC6283083

[22] Long A Zhang L Zhang Y Jiang B Mao Z Li H Efficacy and safety of rivaroxaban versus low-molecular-weight heparin therapy in patients with lower limb fractures. J Thromb Thrombolysis. (2014) 38(3):299–305. 10.1007/s11239-013-1046-1 24402194

[23] Gao Y Long A Xie Z Meng Y Tan J Lv H The compliance of thromboprophylaxis affects the risk of venous thromboembolism in patients undergoing hip fracture surgery. Springerplus. (2016) 5(1):1362. 10.1186/s40064-016-2724-1 27588255 PMC4990524

[24] Bergqvist D Arcelus JI Felicissimo P. Post-discharge compliance to venous thromboembolism prophylaxis in high-risk orthopaedic surgery: results from the ETHOS registry. Thromb Haemost. (2012) 107(2):280–7. 10.1160/TH11-07-0464 22186771

[25] Yip W Fu H Chen AT Zhai T Jian W Xu R 10 years of health-care reform in China: progress and gaps in universal health coverage. Lancet. (2019) 394(10204):1192–204. 10.1016/S0140-6736(19)32136-1 31571602

[26] Clexane [package insert]. Greenville, SC: Sanofi-Aventis (2017). Available at: https://www.sanofi.cn/assets/dot-cn/pages/docs/products/prescription-products/clexane-cn-20240928.pdf (Accessed May 05, 2025).

[27] Garcia DA Baglin TP Weitz JI Samama MM. Parenteral anticoagulants: antithrombotic therapy and prevention of thrombosis, 9th ed: American College of Chest Physicians evidence-based clinical practice guidelines. Chest. (2012) 141(2 Suppl):e24S–43. 10.1378/chest.11-2291 22315264 PMC3278070

[28] Rojas L Aizman A Ernst D Acuña MP Moya P Mellado R Anti-Xa activity after enoxaparin prophylaxis in hospitalized patients weighing less than fifty-five kilograms. Thromb Res. (2013) 132(6):761–4. 10.1016/j.thromres.2013.10.005 24521789

[29] Kwong LM. Hip fracture and venous thromboembolism in the elderly. J Surg Orthop Adv. (2004) 13(3):139–48. 15559689

[30] Wilke T Moock J Müller S Pfannkuche M Kurth A. Nonadherence in outpatient thrombosis prophylaxis with low molecular weight heparins after major orthopaedic surgery. Clin Orthop Relat Res. (2010) 468(9):2437–53. 10.1007/s11999-010-1306-8 20333493 PMC2919876

[31] Xu J Chang D Chui J Cao J Negus J. The efficacy and cost-effectiveness of enoxaparin versus rivaroxaban in the prevention of venous thromboembolism following total hip or knee arthroplasty: a meta-analysis. J Orthop. (2022) 30:1–6. 10.1016/j.jor.2022.02.003 35210718 PMC8844751

[32] Kwong LM. Cost-effectiveness of rivaroxaban after total hip or total knee arthroplasty. Am J Manag Care. (2011) 17(1 Suppl):S22–6. 21517652

